# Liver fibrosis as a predictor of liver failure and outcome following ALPPS among patients with primary liver cancer

**DOI:** 10.1038/s41598-024-65924-2

**Published:** 2024-07-09

**Authors:** Junwei Zhang, Lei Zhang, Xiaobo Yang, Yongchang Zheng, Haifeng Xu, Shunda Du, Yilei Mao, Xinting Sang, Haitao Zhao, Yiyao Xu, Xin Lu

**Affiliations:** Department of Liver Surgery, State Key Laboratory of Complex Severe and Rare Diseases, Peking Union Medical College (PUMC) Hospital, Chinese Academy of Medical Sciences and Peking Union Medical College, Beijing, 100730 China

**Keywords:** Liver regeneration, Liver fibrosis, ALPPS, Liver failure, Liver cancer, Cancer, Medical research, Metabolic disorders

## Abstract

The influence of liver fibrosis on the rate of liver regeneration and complications following ALPPS has yet to be fully understood. This study aimed to scrutinize the effects of liver fibrosis on the postoperative complications, and prognosis subsequent to ALPPS. Clinical data were collected from patients with primary liver cancer who underwent ALPPS at Peking Union Medical College Hospital between May 2014 and October 2022. The degree of liver fibrosis was assessed using haematoxylin–eosin staining and Sirius red staining. This study encompassed thirty patients who underwent ALPPS for primary liver cancer, and there were 23 patients with hepatocellular carcinoma, 5 with cholangiocarcinoma, and 2 with combined hepatocellular-cholangiocarcinoma. The impact of severe liver fibrosis on the rate of liver regeneration was not statistically significant (*P* = 0.892). All patients with severe complications belonged to the severe liver fibrosis group. Severe liver fibrosis exhibited a significant association with 90 days mortality (*P* = 0.014) and overall survival (*P* = 0.012). Severe liver fibrosis emerges as a crucial risk factor for liver failure and perioperative mortality following the second step of ALPPS. Preoperative liver function impairment is an important predictive factor for postoperative liver failure.

## Introduction

Primary liver cancer ranks as the fourth most prevalent malignant tumour in China and is the second leading cause of cancer-related deaths. The incidence and mortality rates have exhibited an upwards trend^1–3^. Presently, surgical intervention remains the primary approach for treating primary liver cancer^4^. Nevertheless, owing to the subtle onset of this malignancy, a considerable number of patients are diagnosed at an advanced disease stage, leading to missed opportunities for surgical intervention due to insufficient future liver remnants (FLRs)^5^.

Associating liver partition and portal vein ligation for staged hepatectomy (ALPPS) has emerged as a significant advancement in liver cancer surgery, facilitating FLR growth within a short timeframe. This technique has swiftly gained global adoption among surgeons^6^. ALPPS can be safely applied in patients with colorectal cancer liver metastasis in the absence of liver fibrosis^7^. However, the application of ALPPS in primary liver cancer patients remains a subject of debate^8^. Research indicates that, compared with portal vein embolization, ALPPS may yield a greater resection rate for primary liver cancer^9^. Nevertheless, some studies suggest that primary liver cancer patients may experience slow liver regeneration after ALPPS, leading to elevated rates of postoperative complications and mortality^10,11^.

The success of ALPPS surgery relies fundamentally on the regenerative capacity of the liver^12^. In cases where the pace of liver regeneration falls short of compensating for liver function, hepatectomy may result in liver failure, potentially leading to fatal outcomes. Currently, the impact of liver fibrosis on the speed of liver regeneration remains a matter of debate. Several studies have proposed that fibrotic livers can undergo complete regeneration similar to that of normal livers^13^. Conversely, other research has suggested a negative correlation between the degree of liver fibrosis and liver regeneration^14^. However, further investigations are needed to elucidate the impact and underlying mechanisms of liver fibrosis on liver regeneration and the recovery of liver function. The association between the degree of liver fibrosis and postoperative complications following ALPPS also remains controversial.

In this retrospective study, patient data was obtained from patients treated with ALPPS for primary liver cancer at a single center within Peking Union Medical College Hospital (PUMCH). Liver fibrosis staging was conducted using haematoxylin–eosin staining and Sirius red staining. Subsequently, an in-depth analysis was conducted to explore the associations between the degree of liver fibrosis and variables such as liver regeneration, postoperative complications, and perioperative mortality.

## Methods

### Study design

This study encompasses the clinical data of patients diagnosed with primary liver cancer who underwent ALPPS at PUMCH from May 1, 2014, to October 31, 2022. The inclusion criteria were as follows: (1) For patients with liver fibrosis, the FLR should be less than 40% of the standard liver volume or the FLR (mL)/body weight (kg) should be less than 0.8%. For patients without liver fibrosis, the FLR/SLV should be less than 30% or the FLR (mL)/body weight (kg) should be less than 0.6%. (2) The preoperative Child‒Pugh score ranged from 5 to 7 points. (3) Patients had no evidence of extrahepatic metastasis.

All enrolled patients were histopathologically confirmed to have primary liver cancer postoperatively. General patient information, including sex, height, weight, comorbidities, and other baseline characteristics, was extracted from medical records. ALPPS-related data, such as operation time, intraoperative blood loss, blood transfusion volume, intraoperative hepatic portal occlusion time, and frequency, were collected from surgical records. Paraffin-embedded liver fibrosis staging samples from ALPPS patients were obtained by the Department of Pathology at PUMCH. These samples represented normal liver tissues on the tumour side following the second step of ALPPS. This study received approval from the Ethics Committee of PUMCH, with ethics number I-22PJ-076, and was conducted in accordance with the principles outlined in the Declaration of Helsinki.

### Endpoints and definition

The primary objective of this study was to investigate the correlation between the degree of liver fibrosis and postoperative outcomes, including the rate of liver regeneration, the occurrence of severe complications within 90 days post-surgery, and the 90 days mortality rate. Additionally, a secondary aim was to analyse the association between the degree of liver fibrosis and disease-free survival (DFS) and overall survival (OS) times.

Postoperative complications were defined as surgery-related issues that emerged either during hospitalization or within 90 days postsurgery. The Clavien‒Dindo classification was used to categorize these complications. Post hepatectomy liver failure (PHLF) was analyzed using the definitions established by the International Study Group of Liver Surgery (ISGLS)^15^. Patients who underwent ALPPS were subjected to regular follow-ups every three months. The follow-up assessments included monitoring postoperative complications, recurrence status, and the nature of the postoperative treatments. DFS was defined as the duration from the second operation to the initial detection of disease recurrence. OS represented the interval from the second operation to either the patient's death or the last follow-up.

### Measurement and calculation of liver volume

Prior to the ALPPS procedure, liver volume was measured for all patients. The assessment of liver volume involved a three-dimensional reconstruction method, in which two radiologists performed the measurements. All volume data were postprocessed on the Advantage Workstation 4.1.2 from GE Healthcare.

The FLR was calculated by manually delineating the remaining liver segments on the axial plane. In cases where necessary, any postoperative residuals or other masses, including cysts, portal branches, or bile ducts, were excluded through threshold segmentation techniques. This meticulous process ensured accurate and comprehensive assessments of liver volume changes associated with the ALPPS procedure.

The standard liver volume (SLV) was determined using the Urata formula: SLV (mL) = 758.259 * body surface area (m2) − 124.27^16^. Subsequently, based on the FLR measured before the first and second steps of surgery, the absolute growth of the FLR (mL) and the growth relative to the liver volume before the first step (expressed as relative growth in percentage) were calculated. The absolute rate and relative rate of liver volume growth were computed by dividing the absolute growth (mL) and relative growth (%) of the FLR by the interval between the first step of ALPPS and the time point of CT measurement preceding the second step. These calculations provided a comprehensive understanding of the dynamic changes in liver volume associated with the ALPPS procedure over specific time intervals.

### ALPPS operation process

The ALPPS procedure encompasses two surgical approaches: open ALPPS and laparoscopic-assisted ALPPS. In open ALPPS, both the first and second steps of the procedure are conducted via laparotomy^17^. In laparoscopic-assisted ALPPS, the first step is carried out with laparoscopic assistance, and the second step involves either open surgery or both steps performed with laparoscopic assistance.

### Grading of liver fibrosis

Wax blocks containing liver tissues were procured from specimens removed after the second step of surgery. Slices (5 μm) of normal liver tissue from the specimens were prepared, and additional haematoxylin–eosin (HE) staining and Sirius red staining was then performed to assess the degree of liver fibrosis. The grading of liver fibrosis was determined, and patients were categorized into two groups, the nonsevere liver fibrosis group (grades 0–4) and the severe liver fibrosis group (grades 5–6), based on the Ishak score^18^.

### Statistical methods

Continuous variables are presented as medians with interquartile ranges (IQRs), while categorical variables are expressed as percentages. The Mann‒Whitney U test was used for the comparison of continuous variables between two groups, and the Pearson chi-square test was used for the comparison of categorical data. One-way analysis of variance was applied for comparisons involving multiple groups. Disease-free survival time and overall survival time were calculated using the Kaplan‒Meier method, with statistical assessments conducted using the log-rank test. A bilateral *P* value less than 0.05 was considered to indicate statistical significance. Because the sample size was too small, multivariate analysis was not carried out. The statistical analyses were performed using IBM SPSS 27.0 statistical software, and GraphPad Prism 9.0 software was used for data visualization.

### Ethical approval

This study received approval from the Ethics Committee of Peking Union Medical College Hospital with ethics number I-22PJ-076. The need to obtain informed consent was waived by the Ethics Committee of Peking Union Medical College Hospital.

## Results

### Basic information of all cases

The study comprised a total of 30 patients diagnosed with primary liver cancer who underwent ALPPS. Among them, 23 patients had hepatocellular carcinoma, 5 patients had cholangiocarcinoma, and 2 patients had mixed hepatocellular carcinoma. The cohort consisted of 24 males and 6 females, with a median age of 54 years (IQR 45, 61). Six patients had comorbidities, with 2 (6.67%) having hypertension and 4 (13.33%) having diabetes. The Eastern Cooperative Oncology Group (ECOG) score for all patients was 0 or 1, and the Child‒Pugh score ranged from 5 to 7. HBV infection was present in 25 patients (73.33%), and one patient had HCV infection. The tumours in 26 patients (86.67%) were singular, and the tumours in 28 patients (93.33%) exceeded 5 cm in size. Eight patients (26.67%) underwent preoperative treatment, with 4 (13.33%) receiving local treatment (Table 1).

**Table 1:** Demographic data, tumor characteristics and perioperative outcomes of the 30 patients.

Variables	All patients (n = 30)
Gender, (n, %)	
Male	24 (80)
Female	6 (20)
Age (median, quartile)	54 (45, 61)
Type of tumor (n, %)	
Hepatocellular carcinoma	23 (76.67)
Cholangiocarcinoma	5 (16.67)
Mixed cell carcinoma	2 (6.67)
BMI (median, quartile)	23.34 (22.21, 24.77)
Comorbidity (n, %)	
Hypertension	2 (6.67)
Diabetes	4 (13.33)
HBV infection (n, %)	
Yes	25 (83.33)
No	5 (16.67)
HCV infection (n, %)	
Yes	1 (3.33)
No	29 (96.67)
Grading of liver fibrosis (n, %)	
0	1 (3.33)
1	3 (10.00)
2	2 (6.67)
3	7 (23.33)
4	3 (10.00)
5	5 (16.67)
6	9 (30.00)
AFP (n, %) (μg/mL)	
< 400	12 (40.00)
≥ 400	18 (60.00)
Total number lesion (n, %)	
1	26 (86.67)
> 1	4 (13.33)
Largest liver lesion,cm (n, %) (cm)	
< 5	2 (6.67)
≥ 5	28 (93.33)
ECOG score (n, %)	
0	21 (70.00)
1	9 (30.00)
Child–Pugh score (n, %)	
5	15 (50.00)
6	13 (43.33)
7	2 (6.67)
Treatment before stage I (n, %)	
Local therapy	4 (13.33)
Systematic therapy	4 (13.33)
No therapy	22 (73.33)

BMI, body mass index; HBV, hepatitis B virus; HCV, hepatitis C virus; AFP, alpha-fetoprotein; ECOG, eastern cooperative oncology group.

### Grouping of liver fibrosis in patients with ALPPS

**Figure 1:**
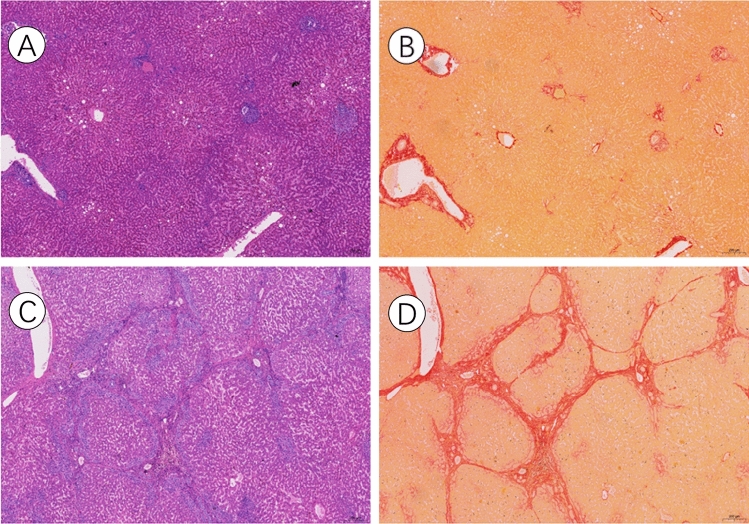
Hematoxylin Eosin (HE) staining and sirius red staining in patients with non-severe fibrosis and severe liver fibrosis. There was no fibrous hyperplasia in HE staining of patients with non-severe liver fibrosis in (**A**). Sirius red staining in patients with non-severe liver fibrosis also did not show fiber regeneration in (**B**). HE staining of patients with severe liver fibrosis showed obvious bridging fibrosis in (**C**). Sirius red staining of patients with severe liver fibrosis showed significant bridging fibrosis and regenerative nodule formation, and red showed fibers in (**D**). The scale is 200 μm.

Among the study participants, 16 patients were classified into the nonsevere liver fibrosis group (Ishak score 0–4). The HE staining and Sirius red staining results of these patients revealed mild fibrosis or an absence of fibrosis in the portal area (Fig. 1 A, B). On the other hand, 14 patients belonged to the severe liver fibrosis group (Ishak score 5–6). For the severe liver fibrosis group, the results of the HE staining and Sirius red staining showed pronounced bridging fibrosis, including central vein and portal area bridging fibrosis, as well as portal area and portal area bridging fibrosis, along with the formation of regenerative nodules (Fig. 1C,D). These histological assessments provided a clear differentiation between the nonsevere and severe liver fibrosis groups based on the Ishak scoring system. Between the nonsevere and severe liver fibrosis groups, there was no difference in the preoperative status before the first step of surgery (Table 2).

**Table 2:** Comparison table of severe liver fibrosis group and non-severe liver fibrosis of the perioperative period.

	Severe liver fibrosis group (n = 14)	Non-severe liver fibrosis group (n = 16)	*P* value
Age (years)	56.00 (48.00, 63.00)	54.00 (43.00, 58.00)	0.224
Gender (male) (%)	9 (92.90%)	11 (68.80%)	0.175
Hepatocellular carcinoma (Yes) (%)	12 (85.70%)	11 (68.80%)	0.399
White blood cells (× 10^9^/L)	4.82 (4.12, 7.35)	6.20 (4.67, 7.14)	0.473
Hemoglobin (g/L)	143.5 (131.00, 152.00)	148 (139.00, 151.00)	0.886
Platelets (× 10^9^/L)	170.5 (124.5, 190.0)	191 (126, 321)	0.552
Total bilirubin (μmol/L)	16.8 (12.88, 19.93)	14.10 (11.40, 19.70)	0.208
Direct bilirubin (μmol/L)	5.75 (5.30, 8.25)	4.90 (3.20, 9.30)	0.131
Albumin (g/L)	42.00 (36.00, 44.25)	40.00 (37.00, 44.00)	0.667
Prothrombin time (s)	12.75 (12.08, 13.65)	12.10 (11.80, 12.70)	0.110
Child–Pugh score	6.0 (5.0–6.0)	5 (5.0–6.0)	0.464
Creatinine (μmol/L)	73.00 (65.5, 79.50)	65.00 (56.00, 75.00)	0.101
INR	1.10 (1.04, 1.17)	1.01 (0.98, 1.10)	0.058
Cholinesterase (kU/L)	5.45 (4.48, 6.55)	6.40 (5.40, 7.90)	0.172

INR, international normalized ratio.

### The effect of liver fibrosis on ALPPS surgery

The median operation time for the first step of ALPPS was 287.50 min (IQR 234.75, 328.75), and for the second step, it was 222.50 min (IQR 205.00, 246.25). In the first step, the median blood loss was 375.00 mL (IQR 137.50, 600.00), with a median blood transfusion of 2 units of red blood cells (IQR 0, 4). For the second step, the median blood loss was 500 mL (IQR 300, 800), and the median blood transfusion volume was 3.75 units (IQR 2, 4) of red blood cells. A total of 9 patients opted for laparoscopic-assisted surgery in the first step, 1 patient was converted to open surgery due to surgical challenges, and 2 patients underwent laparoscopic surgery in the second step (Table 3).

**Table 3:** Surgical outcome for stage I and stage II of ALPPS.

	Stage I (n = 30)	Stage II (n = 30)
Surgery time (min)	287.50 (234.75, 328.75)	222.50 (205.00, 246.25)
Blood loss (mL)	375.00 (137.50, 600.00)	500.00 (300.00, 800.00)
Transfusion volume (U)	2 (0, 4)	3.75 (2, 4)
Laparoscopic surgery (Yes)	8 (26.67%)	2 (6.67%)
Hepatic portal occlusion (Yes)	8 (26.67%)	3 (10.00%)
Postoperative return to ICU (Yes)	2 (6.67%)	5 (16.67%)

There were no differences in operation time, blood loss, blood transfusion, or hepatic portal occlusion time between the severe liver fibrosis group and the nonsevere liver fibrosis group in the first step of ALPPS (Table 4). Further analysis of patients undergoing the second step of ALPPS revealed that the median operation time in the nonsevere liver fibrosis group was 233.00 min (IQR 210.00, 257.50) compared to 210.00 min (IQR 190.00, 227.50) in the severe liver fibrosis group, which was a statistically significant difference (*P* = 0.038). However, there were no differences between the two groups in terms of bleeding, blood transfusion, laparoscopic surgery, or hepatic portal occlusion in the second step of ALPPS (Table 5). All patients in this study successfully completed the two-step resection. The median time interval between the two steps was 12.35 days (IQR 8.50, 20.29). The residual liver volume increased by 202.5 mL (IQR 104, 269), with a median daily increase of 12.34 mL (IQR 8.50, 20.29) (Table 6).

**Table 4:** Comparison of conditions between severe liver fibrosis group and non-severe liver fibrosis group for stage I.

	Severe liver fibrosis group (n = 14)	Non-severe liver fibrosis group (n = 16)	*p* value
Surgery time (min)	297.5 (253.50, 325.00)	272.50 (227.50, 353.75)	0.667
Blood loss (mL)	400.00 (300.00, 525.00)	250.00 (62.50, 800.00)	0.697
Transfusion volume (U)	2.5 (2, 4)	1 (0, 4)	0.918
Laparoscopic surgery (Yes)	2 (14.29%)	6 (37.50%)	0.226
Hepatic portal occlusion (Yes)	4 (28.57%)	4 (25.00%)	1.000

**Table 5:** Comparison of conditions between Severe Liver Fibrosis Group and Non-Severe Liver Fibrosis Group for stage II.

	Severe liver fibrosis group (n = 14)	Non-severe liver fibrosis group (n = 16)	*p* value
Surgery time (min)	210.00 (190.00, 227.50)	233.00 (210.00, 257.50)	0.038
Blood loss (mL)	600 (300, 1000)	500 (400, 775)	0.667
Transfusion volume (U)	3.75 (2, 4)	3 (2, 4)	0.886
Laparoscopic surgery (Yes)	3 (21.43%)	0 (0.00%)	0.228
Hepatic portal occlusion (Yes)	2 (14.29%)	1 (6.25%)	1.000

**Table 6:** Volumetric measurements in ALPPS patients.

Factors	Median (interquartile range)
Stage interval , days	14.00 (12.00, 18.05)
Standard liver volume (Urata Formula), mL	1277.60 (1217.83, 1316.21)
Absolute increase in future liver volume, mL	202.50 (104.00, 269.00)
Relative increase in future liver volume (%)	57.91 (30.39, 80.51)
Increase in furure liver volume/standard liver volume (%)	16.49 (9.26, 21.77)
Future liver volume growth rate, mL/day	12.34 (8.50, 20.29)
Relative residual liver volume growth rate, %/day	3.89 (2.29, 10.33)
Future liver volume/standard liver volume growth rate, %/day	1.03 (0.68, 1.74)

### The effect of liver fibrosis on liver regeneration after the first step of ALPPS

**Figure 2:**
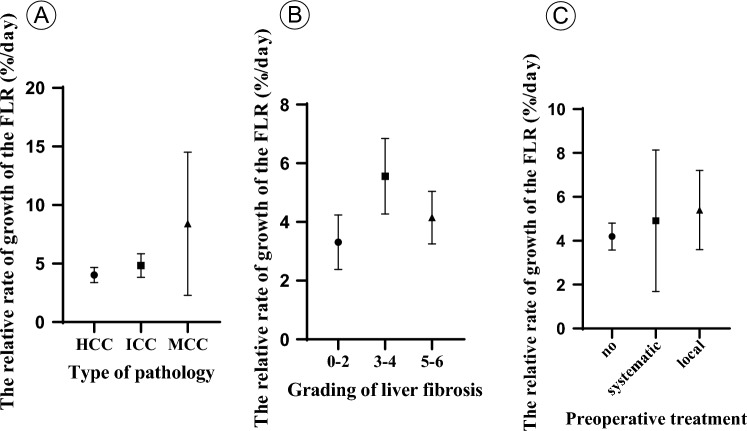
Effects of pathological type, liver fibrosis grade and preoperative treatment on liver rate after ALPPS. The effect of pathological type on liver regeneration rate in (**A**), (*P* = 0.222, one-way ANOVA test). The effect of liver fibrosis grading on liver regeneration rate in (**B**), (*P* = 0.892, one-way ANOVA test). The effect of preoperative treatment on liver regeneration rate in (**C**) (*P* = 0.791, one-way ANOVA test). *Abbreviations* HCC, Hepatocellular carcinoma; ICC, Intrahepatic cholangiocarcinoma; MCC, mixed cell carcinoma.

When examining the relationships between pathological type, liver fibrosis, preoperative ALPPS treatment, and the rate of residual liver volume growth, no statistically significant effects on the liver regeneration rate were observed. However, among the different pathological types, hepatocellular carcinoma patients exhibited slower proliferation than cholangiocarcinoma and mixed cell carcinoma patients did (Fig. 2A). Within the liver fibrosis group, there was a decreasing trend in the liver regeneration rate among patients with severe liver fibrosis (Fig. 2B). Preoperative treatment had a minimal impact on the rate of liver regeneration (Fig. 2C).

Regarding the association between the degree of liver fibrosis and postoperative complications of ALPPS, eight cases of Grade I and Grade II complications, including ascites, diarrhoea, bile leakage, coagulation dysfunction, and abdominal infection, occurred after the first step. Two cases of Grade IIIa complications involving reactive pleural effusion and surgery-related celiac artery bleeding were noted. Additionally, two cases of grade IV complications involving transient acute renal failure were observed. Overall, the incidence of severe complications (≥ grade IIIb) after the first step of ALPPS was 6.67% (2/30) Six patients experienced post hepatectomy liver failure (PHLF) after the stage II operation. According to the ISGLS criteria, there were two patients in each of the ABC grades of hepatic failure.

Following the second step of ALPPS, 12 cases of Grade I and Grade II complications were reported, predominantly including ascites, bile leakage, and coagulation dysfunction. There were four cases of severe complications, resulting in an incidence of 13.33% (4/30), and these severe cases were mainly due to liver failure. Two patients died within 30 days after surgery due to liver failure, while the other two patients died within 90 days after surgery despite an improvement in liver function following treatment (Table 7).

**Table 7:** Complications of ALPPS after stage I and stage II.

Grade	Stage I	Stage II
I	Self-resolving intra-abdominal fluid accumulation (n = 2); Diarrhea (n = 1); Bile leak (n = 1)	Self-resolving intra-abdominal fluid accumulation (n = 2); Bile leak (n = 3)
II	Coagulation dysfunction (n = 1); Infection (n = 3)	Coagulation dysfunction (n = 3); Abnormal liver function (n = 2); Intra-abdominal fluid requiring diuretic treatment (n = 2)
III	Pleural effusion requiring local anesthesia-guided drainage (n = 1); Intra-abdominal bleeding requiring local anesthesia-guided interventional embolization (n = 1)	
IV	Acute renal failure (n = 2)	Liver failure (n = 2)
V		Liver failure with coagulation dysfunction (n = 1) Liver failure with lower gastrointestinal bleeding (n = 1)

All the patients who experienced severe complications after the first step of ALPPS and those who encountered severe complications after the second step of ALPPS were included in the severe liver fibrosis group. Hence, there appears to be a close association between severe liver fibrosis and the occurrence of severe complications following ALPPS.

### The relationship between the degree of liver fibrosis and perioperative death of ALPPS

Thirty days after the second step of ALPPS, two patients had died, for a mortality rate of 6.67% (2/30). Both fatalities were attributed to postoperative liver failure. Within 90 days after the second operation, five patients passed away—four patients with hepatocellular carcinoma and one patient with cholangiocarcinoma. Among the hepatocellular carcinoma patients, all four deaths were due to postoperative liver failure, while the patient with cholangiocarcinoma succumbed to postoperative metastasis.

This study further compared individuals who died and those who did not die within 90 days after ALPPS, focusing on preoperative status before the first step of surgery. In patients who died during the perioperative period, platelet counts (126 vs. 179 × 10^9^/L, *P* = 0.049) and cholinesterase levels (4.2 vs. 6.2 kU/L, *P* = 0.044) were lower than those in patients who did not die. Furthermore, total bilirubin (19.40 vs. 14.25 μmol/L, *P* = 0.037) and direct bilirubin (9.00 vs. 5.30 μmol/L, *P* = 0.013) were greater in patients who died than in those who did not (Table 8). All five patients who died within 90 days after the operation exhibited evident severe liver fibrosis, and the proportion of patients with severe liver fibrosis was greater than that in the nonsurviving group.

**Table 8:** Comparison table of cases of death and non-death within 90 days of the perioperative period.

	Non death cases (n = 25)	Death cases (n = 5)	*p* value
Age (years)	54.00 (43.50, 57.50)	61.0 (55.00, 63.50)	0.085
Gender (male) (%)	19 (76.00%)	5 (100.00%)	0.553
Hepatocellular carcinoma (Yes) (%)	19 (76.00%)	4 (80.00%)	1.00
White blood cells (× 10^9^/L)	5.65 (4.45, 7.17)	4.86 (3.24, 6.49)	0.335
Hemoglobin (g/L)	148 (139.25, 151.75)	140 (124.50, 159.50)	0.416
Platelets (× 10^9^/L)	179 (133.75, 225.25)	126 (99.50, 162.00)	0.049
Total bilirubin (μmol/L)	14.25 (11.68, 19.10)	19.40 (16.80, 22.85)	0.037
Direct bilirubin (μmol/L)	5.30 (3.75, 6.05)	9.00 (6.85, 11.55)	0.013
Albumin (g/L)	41.00 (37.25, 44.75)	42.0 (34.5, 43.5)	0.666
Prothrombin time (s)	12.25 (12.00, 12.85)	13.5 (12.0, 14.5)	0.065
Child–Pugh score	5 (5.0–6.0)	6.0 (6.0–6.5)	0.022
Creatinine (μmol/L)	72.00 (57.25, 75.75)	66.0 (63.50, 74.00)	0.872
INR	1.05 (1.01, 1.11)	1.17 (1.06, 1.21)	0.031
Cholinesterase (kU/L)	6.2 (5.4, 7.35)	4.5 (3.0, 5.70)	0.044
Severe liver fibrosis (n, %)	9 (36%)	5 (100%)	0.014

INR, international normalized ratio.

### The relationship between the degree of liver fibrosis and the prognosis of ALPPS patients

**Figure 3:**
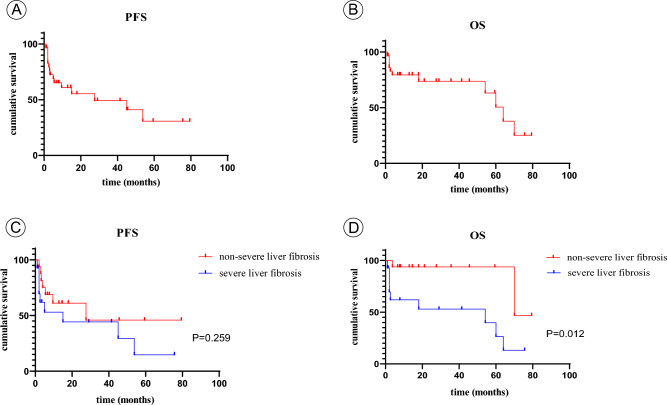
Survival curves of disease-free survival time and overall survival time in patients with ALPPS. There was no difference in disease-free survival between the severe liver fibrosis group and the non-severe liver fibrosis group in (**C**) (*P* = 0.259), while the overall survival time of the severe liver fibrosis group was worse than that of the non-severe liver fibrosis group in (**D**) (*P* = 0.012).

The median follow-up time for all patients included in this study was 16.25 months (IQR 5.85, 47.74). The median disease-free survival time was 22.15 months, and the median overall survival time was 64 months (Fig. 3A,B). During the regular follow-up, 15 patients experienced tumour recurrence. Due to the extended time span for patient enrolment in the study, as well as variations in recurrence status and economic conditions, the treatment measures taken after recurrence varied. Specifically, 5 patients received oral targeted drugs, 4 patients underwent radiofrequency ablation, 4 patients underwent immunotherapy and targeted drug therapy, and 2 patients underwent surgical treatment again due to recurrence.

The recurrence rate for all patients at 1 year was 33.3%. The 1 year survival rate was 60%. No significant difference was observed in disease-free survival time between the severe liver fibrosis group and the nonsevere liver fibrosis group (*P* = 0.259). However, the overall survival time of the severe liver fibrosis group was worse than that of the nonsevere liver fibrosis group (*P* = 0.012) (Fig. 3C,D).

## Discussion

Hepatectomy remains the preferred treatment for primary liver cancer patients^5^. However, its application is often limited by insufficient FLR, and liver failure emerges as a significant postoperative complication^19^. ALPPS has emerged as a potential solution, promoting rapid FLR growth in a short time, which is particularly beneficial for long-term survival in colon cancer liver metastasis patients^7^. Despite its success in certain cases, the application of ALPPS in primary liver cancer patients, often complicated by liver fibrosis and impaired liver function, remains controversial^14^.

This retrospective study, encompassing 30 primary liver cancer patients undergoing ALPPS, delves into the impact of liver fibrosis on post-ALPPS liver regeneration. Among the patients, 14 exhibited severe liver fibrosis (Ishak score 5 and 6). Previous studies have shown that the degree of liver fibrosis has a negative effect on liver regeneration in cirrhotic patients undergoing portal vein embolization (PVE)^20,21^. However, results for ALPPS patients have been controversial. Vennarecci et al.^22^ demonstrated that ALPPS induced considerable and comparable FLR growth in hepatocellular carcinoma (HCC) patients with liver cirrhosis and in patients with colorectal liver metastases (CRLM) and cholangiocarcinoma (CC) with normal liver parenchyma. On the other hand, Wang et al.^23^ reported that the FLR hypertrophy rate negatively correlated with the severity of liver fibrosis and cirrhosis. Our results indicated that severe liver fibrosis did not prevent liver regeneration after ALPPS, although further investigation is warranted in understanding the regenerated liver’s functional capacity. Notably, no significant influence of pathological type or preoperative treatment on the speed of liver regeneration was observed in this study.

The controversy surrounding ALPPS in primary liver cancer arises from its high perioperative complication and mortality rates^11^. Two patients in this study experienced severe complications, primarily transient renal failure, after the first step. Chronic liver injury had been reported as a significant risk factor for postoperative renal failure in extensive hepatectomies^24^. Notably, this study established an association between severe liver fibrosis and renal failure after the first step of ALPPS^25^. The occurrence of renal failure following the initial phase of ALPPS may be linked to liver injury subsequent to portal vein ligation. Further exploration and understanding of the associations between severe liver fibrosis and renal complications are crucial for refining the safety and applicability of ALPPS in the management of primary liver cancer^26,27^.

Notably, liver failure constituted the primary cause of death in the perioperative period of ALPPS. Of the four patients who experienced liver failure, all exhibited severe liver fibrosis, suggesting a potential link between severe liver fibrosis and impaired liver function, contributing to the development of liver failure. The question of whether normal function can be restored after regeneration in a liver fibrosis case remains a subject requiring further investigation. Our results indicated that severe liver fibrosis did not prevent liver regeneration after ALPPS, although further investigation is warranted to understand the functional capacity of the regenerated liver. Some studies have shown that the volume of the liver remnant (LR) calculated by CT volumetry does not equate to the functional capacity of the LR as determined by liver scintigraphy during liver regeneration^28^. This discrepancy may explain why patients with liver cirrhosis, despite having sufficient liver volume, still experienced post hepatectomy liver failure.

In this study, five patients succumbed within 90 days after surgery, resulting in a mortality rate of 16.7%. Strikingly, all these patients exhibited severe liver fibrosis, underscoring the significance of severe liver fibrosis as a critical risk factor for perioperative death in the context of ALPPS. The meticulous consideration of patient indications emerges as a pivotal factor in enhancing the safety of ALPPS procedures^29^. Furthermore, this study suggests that certain preoperative indicators, including thrombocytopenia, elevated bilirubin, decreased cholinesterase, and a high Child‒Pugh score during the initial step of ALPPS, play a crucial role in predicting 90 days mortality after the procedure. These findings highlight the importance of a comprehensive preoperative assessment to identify potential risk factors, ultimately contributing to improved patient outcomes and safety in the context of ALPPS procedures.

To further validate the significance of fibrosis degree as a prognostic factor, we utilized datasets from GSE14520 and The Cancer Genome Atlas (TCGA) to examine the correlation between liver fibrosis markers and patient prognosis^30^. Our analysis revealed that high expression of COL6A3 was associated with poorer prognosis in HCC patients from GSE14520 (Supplementary Fig. 1), and overexpression of MMP-9 was linked to poorer prognosis in HCC patients from TCGA (Supplementary Fig. 2). These findings support our conclusion that fibrosis plays a critical role in the prognosis of HCC patients.

This study implies that identifying the degree of liver fibrosis before undergoing ALPPS may contribute to a reduction in the incidence of perioperative complications and lower the risk of mortality. The current gold standard for diagnosing liver fibrosis remains the pathological examination of liver tissue obtained through puncture. However, liver tissue puncture is an invasive procedure, posing challenges for routine clinical use^31^. While diagnostic models such as the FIB-4 score and APRI score have demonstrated some utility in diagnosing liver fibrosis, their predictive accuracy for determining the specific stage of liver fibrosis is considered suboptimal. These models may provide valuable insights into the presence of fibrosis but may not offer the precision needed to ascertain the extent or severity of fibrotic changes in the liver^32^. As a result, there is ongoing exploration and development of alternative diagnostic approaches, such as liver elastography techniques, to enhance the accuracy of liver fibrosis staging^33,34^. Consequently, future research efforts may focus on developing and validating noninvasive techniques for accurately assessing the degree and severity of liver fibrosis before ALPPS procedures, thereby enhancing patient safety and outcomes.

Several limitations in this study should be acknowledged. First, the sample size is relatively small, and the data is derived from a single centre, potentially limiting the generalizability of the findings to the broader population of patients with primary liver cancer. The inclusion of a substantial number of patients with hepatocellular carcinoma might not fully represent the diverse spectrum of primary liver cancer. Being a retrospective study, the data is subject to incompleteness, and loss of follow-up among patients could impact the overall data quality. This study also spans a wide timeframe, and this could have introduced variability due to the evolution of surgical techniques, including the adoption of laparoscopic approaches, and improvement of the learning curve of health care professionals. Additionally, a multifactorial analysis was not conducted in this study due to the small sample size, and further validation with larger sample sizes and multicentre data is warranted for robust confirmation.

Despite these limitations, this retrospective analysis of liver regeneration, postoperative complications, and survival after ALPPS in primary liver cancer patients from a single centre provides valuable insights. In this study, severe liver fibrosis was shown to be a significant risk factor for perioperative complications and death after ALPPS. Furthermore, this study highlights the predictive value of preoperative indicators such as thrombocytopenia, elevated bilirubin, decreased cholinesterase, and a high Child‒Pugh score, for assessing the risks of liver failure and perioperative death following ALPPS.

### Supplementary Information

Supplementary Figure 1.

Supplementary Figure 2.

## Data Availability

The datasets used during the current study are included in this article and its supplementary information files. The datasets are available from the corresponding author on reasonable request.

## References

[1] Anwanwan D, Singh SK, Singh S, Saikam V, Singh R (2020). Challenges in liver cancer and possible treatment approaches. Biochim. Biophys. Acta Rev. Cancer.

[2] Zhou M, Wang H, Zeng X, Yin P, Zhu J, Chen W, Li X (2019). Mortality, morbidity, and risk factors in China and its provinces, 1990–2017: A systematic analysis for the global burden of disease study 2017. Lancet.

[3] Chen W, Zheng R, Baade PD, Zhang S, Zeng H, Bray F, Jemal A (2016). Cancer statistics in China, 2015. CA Cancer J. Clin..

[4] Fu J, Wang H (2018). Precision diagnosis and treatment of liver cancer in China. Cancer Lett..

[5] Villanueva A (2019). Hepatocellular carcinoma. N. Engl. J. Med..

[6] Lang H, de Santibañes E, Schlitt HJ, Malagó M, van Gulik T, Machado MA, Jovine E (2019). 10th anniversary of ALPPS-lessons learned and quo Vadis. Ann. Surg..

[7] Hasselgren K, Røsok BI, Larsen PN, Sparrelid E, Lindell G, Schultz NA, Bjørnbeth BA (2021). ALPPS improves survival compared with TSH in patients affected of CRLM: Survival analysis from the randomized controlled trial LIGRO. Ann. Surg..

[8] Li PP, Huang G, Jia NY, Pan ZY, Liu H, Yang Y, He CJ (2022). Associating liver partition and portal vein ligation for staged hepatectomy versus sequential transarterial chemoembolization and portal vein embolization in staged hepatectomy for HBV-related hepatocellular carcinoma: A randomized comparative study. Hepatobiliary Surg. Nutr..

[9] Chan A, Zhang WY, Chok K, Dai J, Ji R, Kwan C, Man N (2021). ALPPS versus portal vein embolization for hepatitis-related hepatocellular carcinoma: A changing paradigm in modulation of future liver remnant before major hepatectomy. Ann. Surg..

[10] Cai X, Tong Y, Yu H, Liang X, Wang Y, Liang Y, Li Z (2017). The ALPPS in the treatment of hepatitis B-related hepatocellular carcinoma with cirrhosis: A single-center study and literature review. Surg. Innov..

[11] Chia DKA, Yeo Z, Loh SEK, Iyer SG, Madhavan K, Kow AWC (2018). ALPPS for hepatocellular carcinoma is associated with decreased liver remnant growth. J. Gastrointest. Surg..

[12] Kambakamba P, Stocker D, Reiner CS, Nguyen-Kim TD, Linecker M, Eshmuminov D, Petrowsky H (2016). Liver kinetic growth rate predicts postoperative liver failure after ALPPS. HPB (Oxford).

[13] Liu JG, Wang J, Sun W, Zhang JJ, Wang YJ, Shu GM, Lou C (2021). ALPPS in the treatment of liver cancer with insufficient future liver remnant. Hepatobiliary Pancreat. Dis. Int..

[14] D'Haese JG, Neumann J, Weniger M, Pratschke S, Björnsson B, Ardiles V, Chapman W (2016). Should ALPPS be used for liver resection in intermediate-stage HCC?. Ann. Surg. Oncol..

[15] Rahbari NN, Garden OJ, Padbury R, Brooke-Smith M, Crawford M, Adam R, Koch M (2011). Posthepatectomy liver failure: A definition and grading by the International study group of liver surgery (ISGLS). Surgery.

[16] Feng LM, Wang PQ, Yu H, Chen RT, Wang J, Sheng X, Yuan ZL (2017). New formula for predicting standard liver volume in Chinese adults. World J. Gastroenterol..

[17] Zhang J, Xu Y, Yang H, Huang H, Bian J, Jiang S, Sang X (2020). Application of associating liver partition and portal vein ligation for staged hepatectomy for hepatocellular carcinoma related to hepatitis B virus: Comparison with traditional one-stage right hepatectomy. Transl. Cancer Res..

[18] Ishak K, Baptista A, Bianchi L, Callea F, De Groote J, Gudat F, Denk H (1995). Histological grading and staging of chronic hepatitis. J. Hepatol..

[19] van den Broek MA, Olde Damink SW, Dejong CH, Lang H, Malagó M, Jalan R, Saner FH (2008). Liver failure after partial hepatic resection: Definition, pathophysiology, risk factors and treatment. Liver Int..

[20] Huang YL, Huang MC, Chang CI, Yang LH, Wu CJ, Chiu CC, Chen CY (2023). Elevated intramuscular adipose tissue content with a high Ishak fibrosis stage (>3) had a negative effect on liver regeneration in cirrhotic patients undergoing portal vein embolization. Kaohsiung J. Med. Sci..

[21] Denys A, Lacombe C, Schneider F, Madoff DC, Doenz F, Qanadli SD, Halkic N (2005). Portal vein embolization with N-butyl cyanoacrylate before partial hepatectomy in patients with hepatocellular carcinoma and underlying cirrhosis or advanced fibrosis. J. Vasc. Interv. Radiol..

[22] Vennarecci G, Grazi GL, Sperduti I, Busi Rizzi E, Felli E, Antonini M, D'Offizi G (2016). ALPPS for primary and secondary liver tumors. Int. J. Surg..

[23] Wang Z, Peng Y, Hu J, Wang X, Sun H, Sun J, Shi Y (2020). Associating liver partition and portal vein ligation for staged hepatectomy for unresectable hepatitis B virus-related hepatocellular carcinoma: A single center study of 45 patients. Ann. Surg..

[24] Reese T, Kröger F, Makridis G, Drexler R, Jusufi M, Schneider M, Brüning R (2021). Impact of acute kidney injury after extended liver resections. HPB (Oxford).

[25] Tao W, Shi X, Wang G (2018). Acute kidney injury following the first stage of the ALPPS procedure: A case report. Exp. Ther. Med..

[26] Reese T, Fard-Aghaie MH, Makridis G, Kantas A, Wagner KC, Malagó M, Robles-Campos R (2020). Renal impairment is associated with reduced outcome after associating liver partition and portal vein ligation for staged hepatectomy. J. Gastrointest. Surg..

[27] Huang H, Lu X, Yang H, Xu Y, Sang X, Zhao H (2019). Acute kidney injury after associating liver partition and portal vein ligation for staged hepatectomy for hepatocellular carcinoma: Two case reports and a literature review. Ann. Transl. Med..

[28] Tomassini F, D'Asseler Y, Linecker M, Giglio MC, Castro-Benitez C, Truant S, Axelsson R (2020). Hepatobiliary scintigraphy and kinetic growth rate predict liver failure after ALPPS: A multi-institutional study. HPB (Oxford).

[29] Linecker M, Stavrou GA, Oldhafer KJ, Jenner RM, Seifert B, Lurje G, Bednarsch J (2016). The ALPPS risk score: Avoiding futile use of ALPPS. Ann. Surg..

[30] Nwosu ZC, Megger DA, Hammad S, Sitek B, Roessler S, Ebert MP, Meyer C (2017). Identification of the consistently altered metabolic targets in human hepatocellular carcinoma. Cell Mol. Gastroenterol. Hepatol..

[31] Rousselet MC, Michalak S, Dupré F, Croué A, Bedossa P, Saint-André JP, Calès P (2005). Sources of variability in histological scoring of chronic viral hepatitis. Hepatology.

[32] Dong XQ, Wu Z, Zhao H, Wang GQ (2019). Evaluation and comparison of thirty noninvasive models for diagnosing liver fibrosis in chinese hepatitis B patients. J. Viral Hepat..

[33] Seo YS, Kim MY, Kim SU, Hyun BS, Jang JY, Lee JW, Lee JI (2015). Accuracy of transient elastography in assessing liver fibrosis in chronic viral hepatitis: A multicentre, retrospective study. Liver Int..

[34] Singh S, Venkatesh SK, Wang Z, Miller FH, Motosugi U, Low RN, Hassanein T (2015). Diagnostic performance of magnetic resonance elastography in staging liver fibrosis: A systematic review and meta-analysis of individual participant data. Clin. Gastroenterol. Hepatol..

