# Supplementary figures and images for: Liver fibrosis as a predictor of liver failure and outcome following ALPPS among patients with primary liver cancer
Source: Sci Rep. 2024 Jul 9;14:15827. doi: 10.1038/s41598-024-65924-2 (PMC11233615; doi:10.1038/s41598-024-65924-2)

## Overall Survival

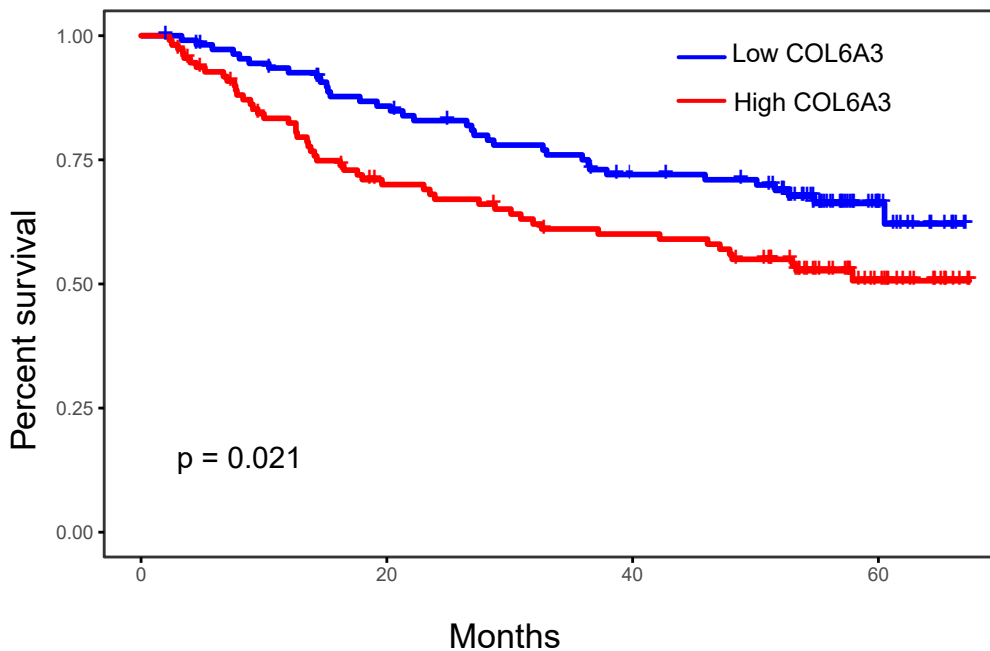

Supplement: Supplementary file 1 — Supplementary Figure 1. [file 41598_2024_65924_MOESM1_ESM.pdf]

# Overall Survival

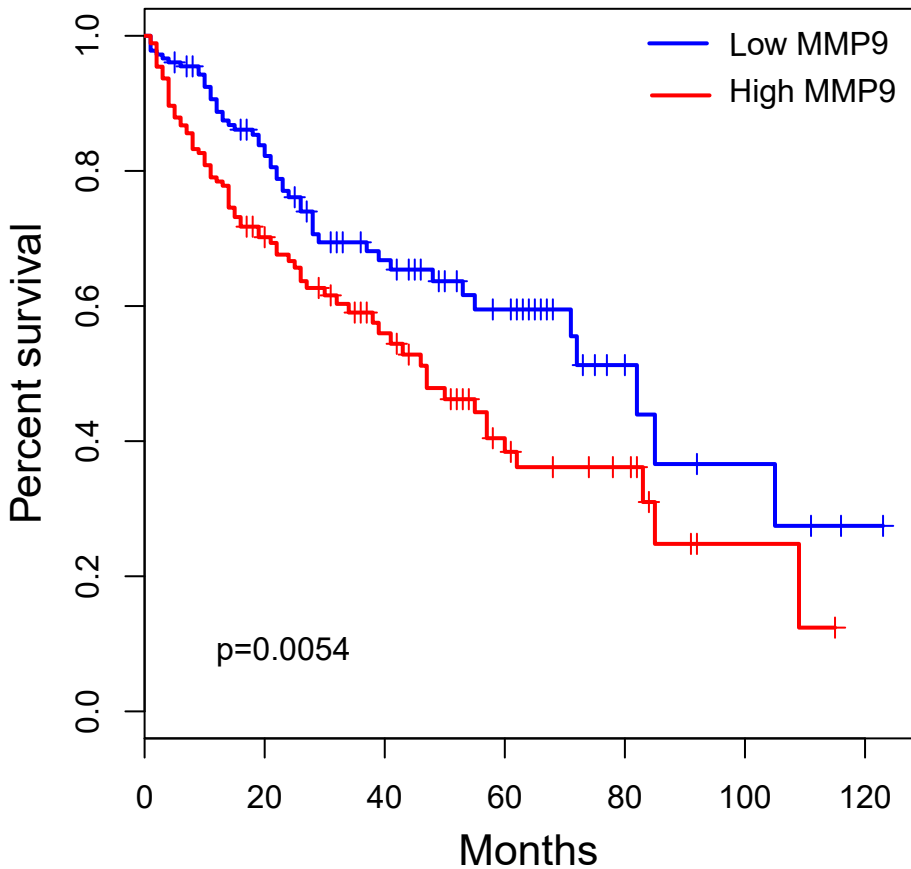

Supplement: Supplementary file 2 — Supplementary Figure 2. [file 41598_2024_65924_MOESM2_ESM.pdf]
